# Supplementary figures and images for: α-synuclein impairs autophagosome maturation through abnormal actin stabilization
Source: PLoS Genet. 2021 Feb 8;17(2):e1009359. doi: 10.1371/journal.pgen.1009359 (PMC7895402; doi:10.1371/journal.pgen.1009359)

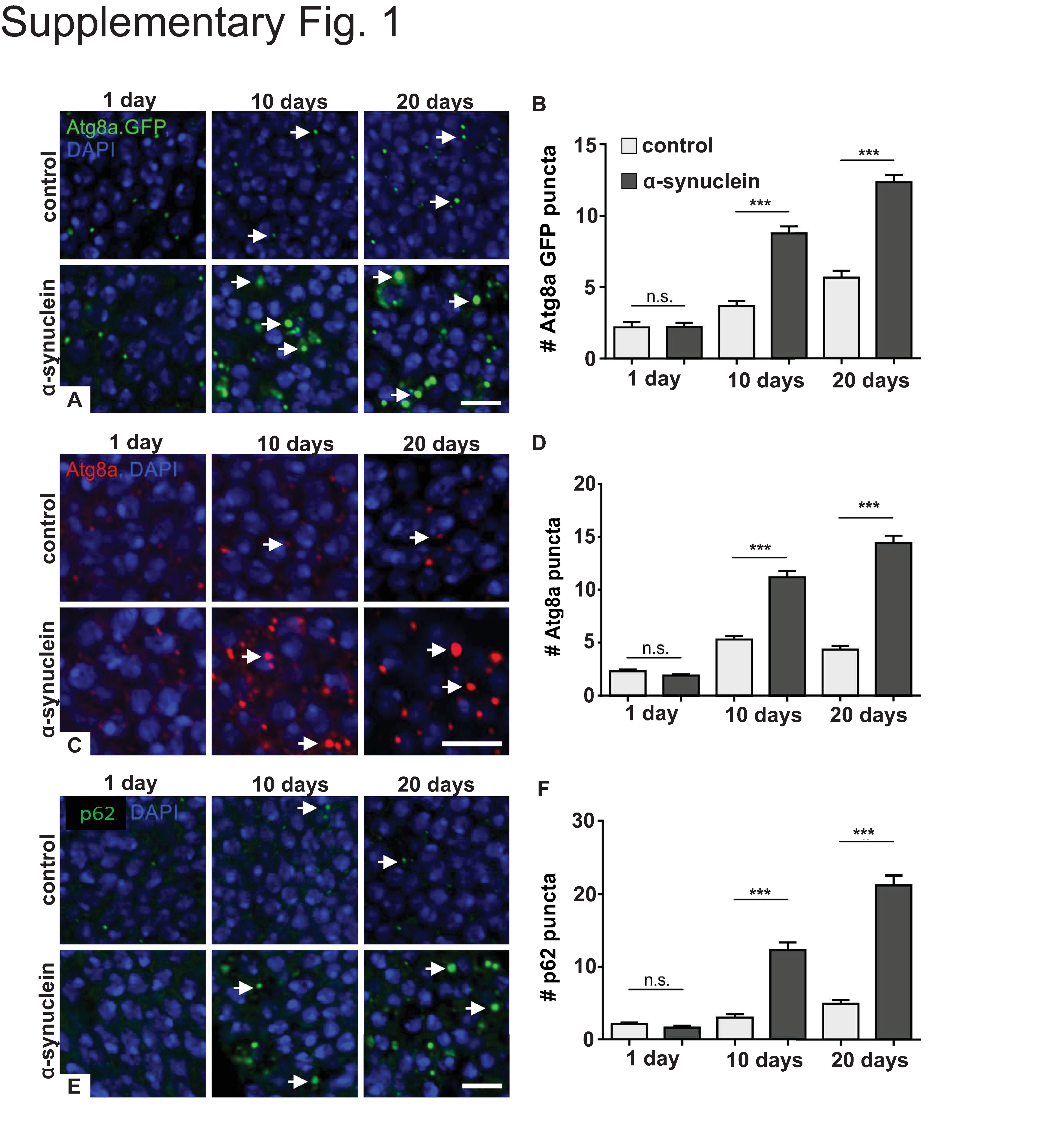

Supplement: S1 Fig — (A) Representative GFP-immunofluorescence images of the anterior medulla and (B) quantification of Atg8a-GFP puncta. Control genotype in (A,B): UAS-Atg8a-GFP/ nSyb-QF2, nSyb-GAL4. (C) Immunofluorescence staining of endogenous Atg8a and (D) quantification of Atg8a-positive puncta. (E, F) Age-dependent accumulation of p62-immunoreactive aggregates as demonstrated by immunofluorescence (E) and (F) quantification of p62-postive puncta. Control genotype in (C-F): nSyb-QF2, nSyb-GAL4/+. Full genotypes are provided for all animals in S1 Text. *** p<0.0001, ANOVA with Tukey’s multiple comparisons test. n.s. not significant. Data are represented as mean ± SEM. n = 6 per genotype. Scale bars are 5 μm. The number of puncta per 500 μm2 is presented. The ages of the flies are indicated in the figure labels. (TIF) [file pgen.1009359.s001.tif]

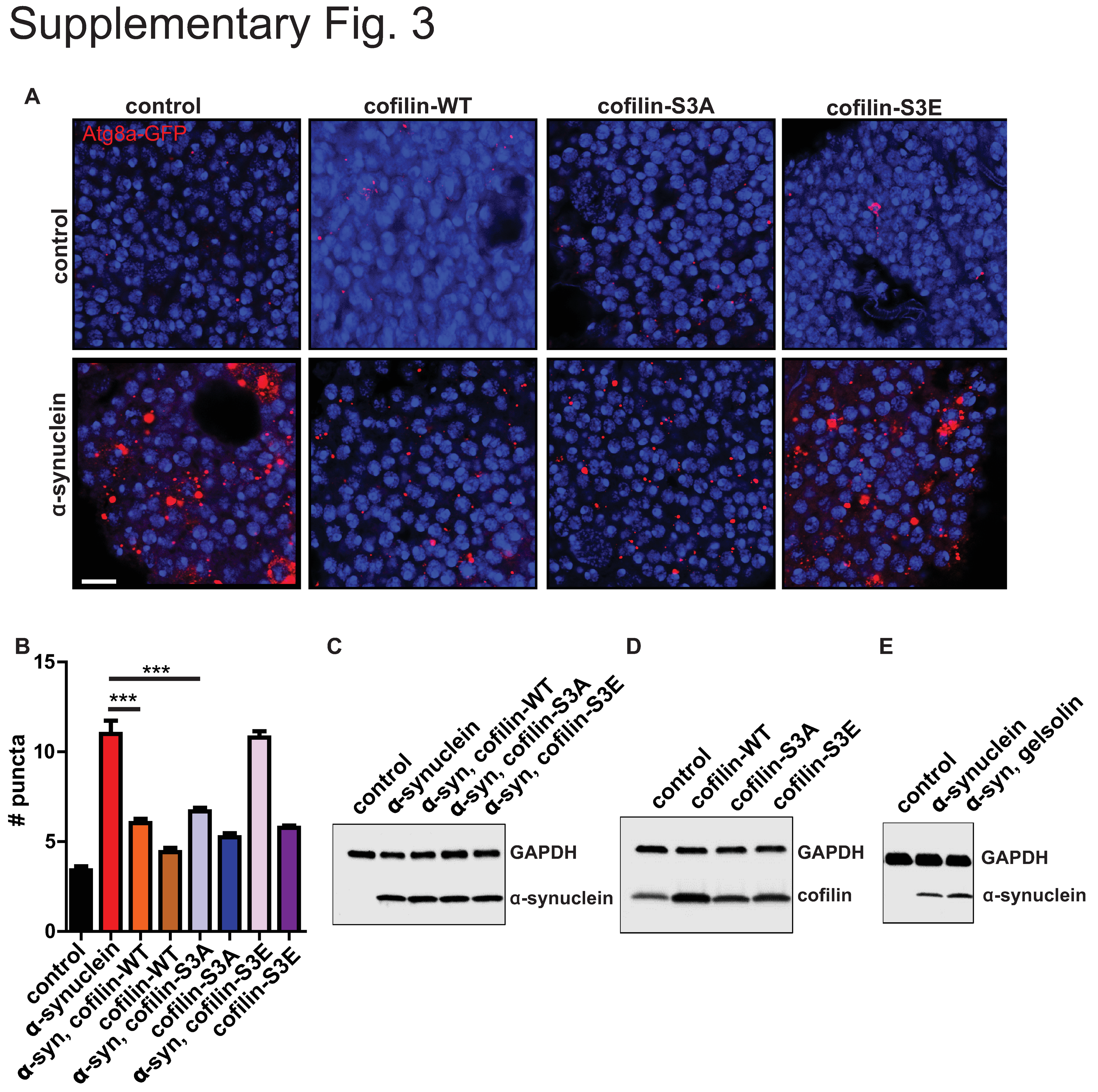

Supplement: S3 Fig — (A) Expression of wild type (WT) and constitutively active cofilinS3A, but not inactive cofilinS3E, rescues α-synuclein-mediated increases in Atg8a-GFP-positive puncta in α-synuclein transgenic flies. (B) Quantification of puncta from (A). (C) Immunoblot of Drosophila head homogenates showing no change in α-synuclein levels among α-synuclein transgenic flies with and without cofilin transgene expression. (D) Immunoblot of Drosophila head homogenates showing comparable cofilin expression levels among all three forms of cofilin. (E) Immunoblot of Drosophila head homogenates showing no change in α-synuclein levels between α-synuclein transgenic flies with and without gelsolin overexpression. All blots were reprobed for GAPDH to illustrate equivalent protein loading. Control genotype in (A,B): nSyb-QF2, nSyb-GAL4, UAS-Atg8a-GFP / +. Control genotype in (C-E): nSyb-QF2, nSyb-GAL4 / +. Full genotypes are provided for all animals in S1 Text. *** p<0.0001, ANOVA with Tukey’s multiple comparisons test. n = 6 per genotype in (A,B). n = 3 per genotype in (C-E). Scale bar is 5 μm (A). The number of puncta per 500 μm2 is presented (A). Flies are 10 days old in (A,B). Flies are 1–3 days old in (C-E). (TIF) [file pgen.1009359.s003.tif]

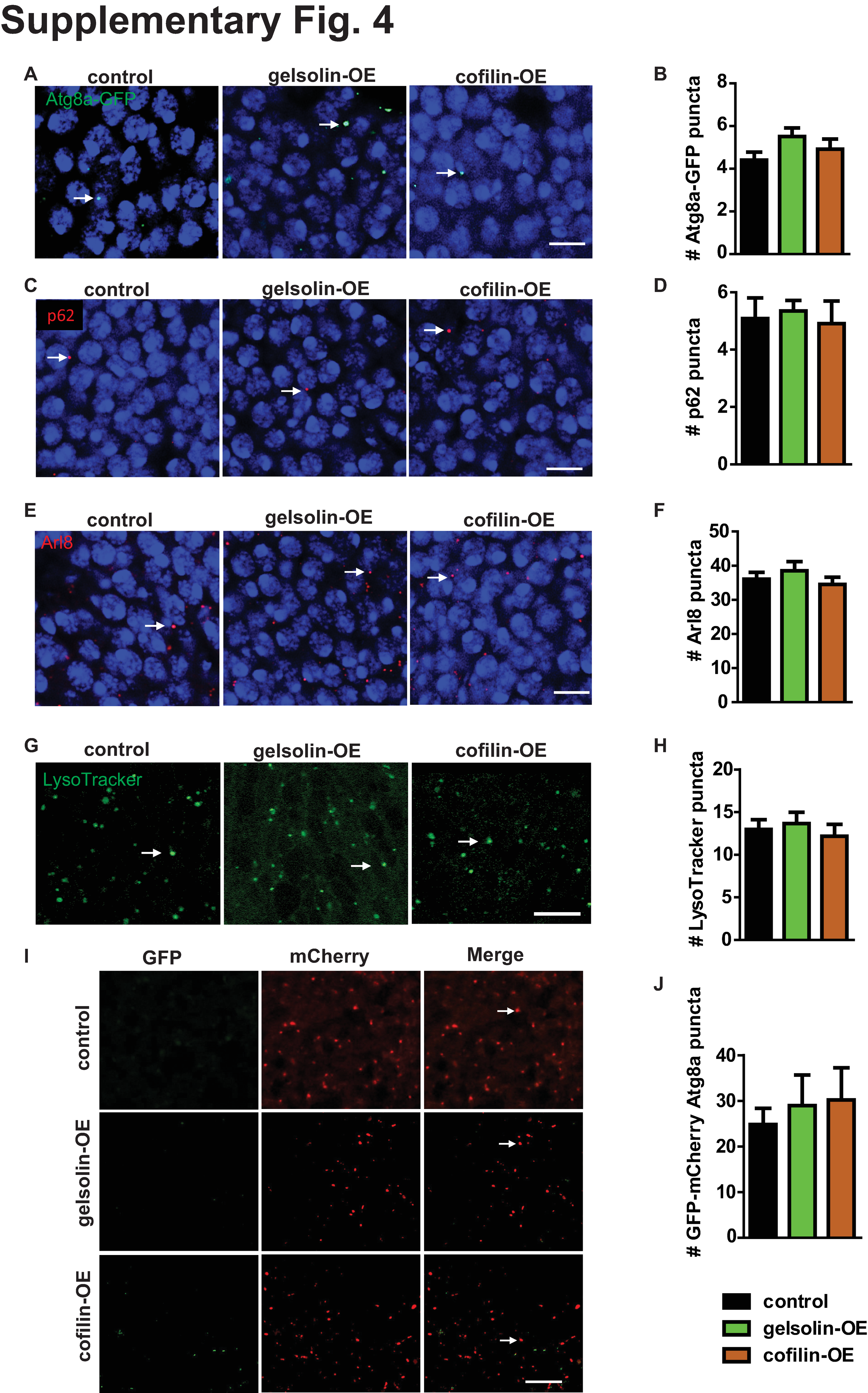

Supplement: S4 Fig — (A-H) No change in the number of Atg8a-GFP (A arrows, B), p62 (C arrows, D), Arl8 (E arrows, F), or LysoTracker (G arrows, H) -positive puncta in flies expressing gelsolin or cofilin. (I) Representative images of brains from flies expressing GFP-mCherry-Atg8a with and without gelsolin or cofilin show no change in the number of mCherry (arrows) or GFP-positive puncta, as quantified by the number of GFP-mCherry-Atg8a dual-positive puncta in (J). Control genotype in (A,B): UAS-Atg8a-GFP/ nSyb-QF2, nSyb-GAL4. Control genotype in (C-H): nSyb-QF2, nSyb-GAL4/+. Control genotype in (I,J): UAS-GFP-mCherry-Atg8a/+; nSyb-QF2, nSyb-GAL4/ +. Full genotypes are provided for all animals in S1 Text. Data are represented as mean ± SEM. n = 6 per genotype in (A-H). n = 4 per genotype in (I,J). Scale bars are 5 μm. The number of puncta per 500 μm2 are presented in (B,D,F,H) and per 1000 μm2 in (J). Flies are 10 days old. (TIF) [file pgen.1009359.s004.tif]

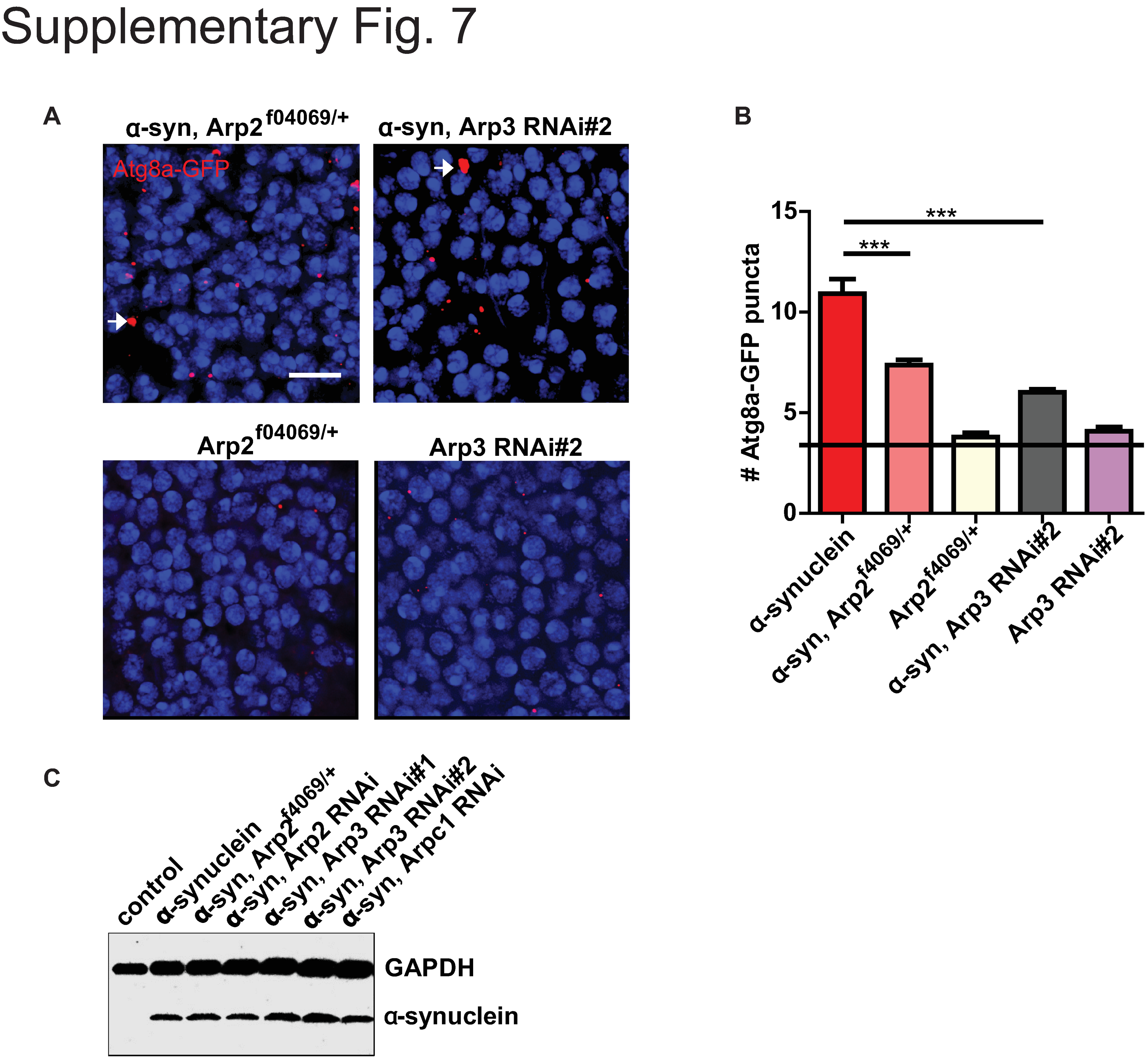

Supplement: S7 Fig — (A) Representative images of Atg8a-GFP (arrows) following knockdown of Arp2 or Arp3 using a heterozygous loss of function Arp2 allele (Arp2f04069 / +) or a confirmatory second Arp3 RNAi line. (B) Quantification of Atg8a-GFP-positive puncta shows a reduced number of Atg8a puncta in α-synuclein transgenic flies. n = 6 per genotype. The solid line in (B) indicates the control value (genotype: nSyb-QF2, nSyb-GAL4, UAS-Atg8a-GFP / +). (C) Immunoblot of Drosophila head homogenates showing no change in α-synuclein levels among α-synuclein transgenic flies with and without Arp2/3 complex member knockdown. The blot is reprobed for GAPDH to illustrate equivalent protein loading. Control genotype in (C): nSyb-QF2, nSyb-GAL4 / +. Full genotypes are provided for all animals in S1 Text. *** p<0.001, ANOVA followed by Tukey’s multiple comparisons test. Data are represented as mean ± SEM. n = 6 per genotype in (A,B) and 3 per genotype in (C). Scale bar is 5 μm (A). The number of puncta per 500 μm2 is presented (B). Flies are 10 days old in (A,B) and 1–3 days old in (C). (TIF) [file pgen.1009359.s007.tif]
