# Supplementary material for: α-synuclein impairs autophagosome maturation through abnormal actin stabilization
Source: PLoS Genet. 2021 Feb 8;17(2):e1009359. doi: 10.1371/journal.pgen.1009359 (PMC7895402; doi:10.1371/journal.pgen.1009359)
Supplement: S1 Text — (DOC) [file pgen.1009359.s009.doc]

**S1 Text. Genotypes for all experiments.**

Fig 1A

Control: *UAS-Atg8a-GFP / nSyb-QF2, nSyb-GAL4*

α-synuclein (α-syn): *QUAS-wild type α-synuclein*, *nSyb-QF2, nSyb-GAL4 / UAS-Atg8a-GFP* Fig 1B, C, E, G, I, J

Control: *nSyb-QF2, nSyb-GAL4 / +*

α-synuclein (α-syn): *QUAS-wild type α-synuclein*, *nSyb-QF2, nSyb-GAL4 / +*

Fig 1F

Control: *Rab2^EYFP^ / + ; nSyb-QF2, nSyb-GAL4 / +*

α-synuclein (α-syn): *Rab2^EYFP^ / + ; QUAS-wild type α-synuclein*, *nSyb-QF2, nSyb-GAL4 / +*

Fig 2

Control: *UAS-GFP-mCherry-Atg8a / + ; nSyb-QF2, nSyb-GAL4 / +*.

α-synuclein: *UAS-GFP-mCherry-Atg8a / + ; QUAS-wild type α-synuclein*, *nSyb-QF2, nSyb-GAL4 / +*

Fig 3A and 3B

α-synuclein: *QUAS-wild type α-synuclein*, *nSyb-QF2, nSyb-GAL4, UAS-Atg8a-GFP / +*

α-syn, gelsolin-OE (overexpression): *QUAS-wild type α-synuclein*, *nSyb-QF2, nSyb-GAL4, UAS-Atg8a-GFP / UAS-Gelsolin*

α-syn, cofilin-OE (overexpression): *QUAS-wild type α-synuclein*, *nSyb-QF2, nSyb-GAL4, UAS-Atg8a-GFP / UAS-cofilin*

Fig 3C-H

α-synuclein: *QUAS-wild type α-synuclein*, *nSyb-QF2, nSyb-GAL4 / +*

α-syn, Gelsolin-OE (overexpression): *QUAS-wild type α-synuclein*, *nSyb-QF2, nSyb-GAL4 / UAS-Gelsolin*

α-syn, cofilin-OE (overexpression): *QUAS-wild type α-synuclein*, *nSyb-QF2, nSyb-GAL4 / UAS-cofilin*

Fig 4

α-synuclein (α-syn): *UAS-GFP-mCherry-Atg8a / + ; QUAS-wild type α-synuclein*, *nSyb-QF2, nSyb-GAL4 / +*

α-syn, gelsolin-OE (overexpression): *UAS-GFP-mCherry-Atg8a / + ; QUAS-wild type α-synuclein*, *nSyb-QF2, nSyb-GAL4 / UAS-Gelsolin*

α-syn, cofilin-OE (overexpression): *UAS-GFP-mCherry-Atg8a / + ; QUAS-wild type α-synuclein*, *nSyb-QF2, nSyb-GAL4 / UAS-cofilin*

Fig 5A and 5B

Control: *UAS-GFP-mCherry-Atg8a / + ; nSyb-QF2, nSyb-GAL4 / +*.

α-synuclein: *UAS-GFP-mCherry-Atg8a / + ; QUAS-wild type α-synuclein*, *nSyb-QF2, nSyb-GAL4 / +*

Fig 5C-F.

Control: *UAS-mtKeima / + ; nSyb-QF2, nSyb-GAL4 / +*.

α-synuclein (α-syn): *UAS-mtKeima / + ; QUAS-wild type α-synuclein*, *nSyb-QF2, nSyb-GAL4 / +*

α-syn, gelsolin-OE (overexpression): *UAS-mtKeima / + ; QUAS-wild type α-synuclein*, *nSyb-QF2, nSyb-GAL4 / UAS-Gelsolin*

α-syn, cofilin-OE (overexpression): *UAS-mtKeima / + ; QUAS-wild type α-synuclein*, *nSyb-QF2, nSyb-GAL4 / UAS-cofilin*

Figs 6 and 7

Control: *nSyb-QF2, nSyb-GAL4 / +*

α-synuclein: *QUAS-wild type α-synuclein*, *nSyb-QF2, nSyb-GAL4 / +*

α-syn, gelsolin-OE (overexpression): *QUAS-wild type α-synuclein*, *nSyb-QF2, nSyb-GAL4 / UAS-Gelsolin*

α-syn, cofilin-OE (overexpression): *QUAS-wild type α-synuclein*, *nSyb-QF2, nSyb-GAL4 / UAS-cofilin*

Fig 8A and 8B

Control: *nSyb-QF2, nSyb-GAL4, UAS-Atg8a-GFP / +*

Arp2 RNAi: *UAS-Arp2^JF02785^ / nSyb-QF2, nSyb-GAL4, UAS-Atg8a-GFP*

Arp3 RNAi: *UAS-Arp3^HMS0071^ / nSyb-QF2, nSyb-GAL4, UAS-Atg8a-GFP*

Arpc1 RNAi: *UAS-Arpc1^JF01763^ / nSyb-QF2, nSyb-GAL4, UAS-Atg8a-GFP*

α-synuclein (α-syn): *QUAS-wild type α-synuclein*, *nSyb-QF2, nSyb-GAL4, UAS-Atg8a-GFP / +*

α-syn, Arp2 RNAi: *QUAS-wild type α-synuclein*, *nSyb-QF2, nSyb-GAL4, UAS-Atg8a-GFP / UAS-Arp2^JF02785^*

α-syn, Arp3 RNAi: *QUAS-wild type α-synuclein*, *nSyb-QF2, nSyb-GAL4, UAS-Atg8a-GFP / UAS-Arp2^HMS0071^*

α-syn, Arpc1 RNAi: *QUAS-wild type α-synuclein*, *nSyb-QF2, nSyb-GAL4, UAS-Atg8a-GFP / UAS-Arpc1^JF01763^*

Fig 8C-F

Control: *nSyb-QF2, nSyb-GAL4 / +*

α-synuclein (α-syn): *QUAS-wild type α-synuclein*, *nSyb-QF2, nSyb-GAL4 / +*

Arp2 RNAi: *UAS-Arp2^JF02785^ / nSyb-QF2, nSyb-GAL4*

Arp3 RNAi: *UAS-Arp3^HMS0071^ / nSyb-QF2, nSyb-GAL4*

Arpc1 RNAi: *UAS-Arpc1^JF01763^ / nSyb-QF2, nSyb-GAL4*

α-synuclein (α-syn): *QUAS-wild type α-synuclein*, *nSyb-QF2, nSyb-GAL4 / +*

α-syn, Arp2 RNAi: *QUAS-wild type α-synuclein*, *nSyb-QF2, nSyb-GAL4 / UAS-Arp2^JF02785^*

α-syn, Arp3 RNAi: *QUAS-wild type α-synuclein*, *nSyb-QF2, nSyb-GAL4 / UAS-Arp2^HMS0071^*

α-syn, Arpc1 RNAi: *QUAS-wild type α-synuclein*, *nSyb-QF2, nSyb-GAL4 / UAS-Arpc1^JF01763^*

S1A and S1B Fig

Control: *UAS-Atg8a-GFP / nSyb-QF2, nSyb-GAL4*

α-synuclein: *QUAS-wild type α-synuclein*, *nSyb-QF2, nSyb-GAL4 / UAS-Atg8a-GFP*

S1C-S1F Fig

Control: *nSyb-QF2, nSyb-GAL4 / +*

α-synuclein: *QUAS-wild type α-synuclein*, *nSyb-QF2, nSyb-GAL4 / +*

S2B Fig

Control: *nSyb-QF2, nSyb-GAL4, UAS-Atg8a-GFP / +*

EGFP: *UAS-EGFP* / + ; *nSyb-QF2, nSyb-GAL4, UAS-Atg8a-GFP*

ß-galactosidase (ß-gal): *UAS-lacZ* / + ; *nSyb-QF2, nSyb-GAL4, UAS-Atg8a-GFP*

S2D-S2G Fig

Control: *nSyb-QF2, nSyb-GAL4 / +*

EGFP: *UAS-EGFP* / + ; *nSyb-QF2, nSyb-GAL4 / +*

ß-galactosidase (ß-gal): *UAS-lacZ* / + ; *nSyb-QF2, nSyb-GAL4 / +*

Figure S2H,I.

Control: *UAS-GFP-mCherry-Atg8a / + ; nSyb-QF2, nSyb-GAL4 / +*.

ß-galactosidase (ß-gal): *UAS-lacZ* / *UAS-GFP-mCherry-Atg8a* ; *nSyb-QF2, nSyb-GAL4 / +*

S3A and S3B Fig

Control: *nSyb-QF2, nSyb-GAL4, UAS-Atg8a-GFP / +*

cofilin-WT: *UAS-cofilin^WT^ (wild type) / nSyb-QF2, nSyb-GAL4, UAS-Atg8a-GFP*

cofilin-S3A: *UAS-cofilin^S3A^ / nSyb-QF2, nSyb-GAL4, UAS-Atg8a-GFP*

cofilin-S3E: *UAS-cofilin^S3E^ / nSyb-QF2, nSyb-GAL4, UAS-Atg8a-GFP*

α-synuclein: *QUAS-wild type α-synuclein*, *nSyb-QF2, nSyb-GAL4, UAS-Atg8a-GFP / +*

α-synuclein, cofilin-WT: *QUAS-wild type α-synuclein*, *nSyb-QF2, nSyb-GAL4, UAS-Atg8a-GFP / UAS-cofilin^WT^*

α-synuclein, cofilin S3A: *QUAS-wild type α-synuclein*, *nSyb-QF2, nSyb-GAL4, UAS-Atg8a-GFP / UAS-cofilin^S3A^*

α-synuclein, cofilin S3E: *QUAS-wild type α-synuclein*, *nSyb-QF2, nSyb-GAL4, UAS-Atg8a-GFP / UAS-cofilin^S3E^*

S3C and S3D Fig

Control: *nSyb-QF2, nSyb-GAL4 / +*

α-synuclein: *QUAS-wild type α-synuclein*, *nSyb-QF2, nSyb-GAL4 / +*

cofilin-WT: *UAS-cofilin^WT^ (wild type) / nSyb-QF2, nSyb-GAL4*

cofilin-S3A: *UAS-cofilin^S3A^ / nSyb-QF2, nSyb-GAL4*

cofilin-S3E: *UAS-cofilin^S3E^ / nSyb-QF2, nSyb-GAL4*

α-syn, cofilin-WT: *QUAS-wild type α-synuclein*, *nSyb-QF2, nSyb-GAL4 / UAS-cofilin^WT^*

α-syn, cofilin-S3A: *QUAS-wild type α-synuclein*, *nSyb-QF2, nSyb-GAL4 / UAS-cofilin^S3A^*

α-syn, cofilin-S3E: *QUAS-wild type α-synuclein*, *nSyb-QF2, nSyb-GAL4 / UAS-cofilin^S3E^*

Figure S3E.

Control: *nSyb-QF2, nSyb-GAL4 / +*

α-synuclein: *QUAS-wild type α-synuclein*, *nSyb-QF2, nSyb-GAL4 / +*

α-syn, gelsolin OE (overexpression): *QUAS-wild type α-synuclein*, *nSyb-QF2, nSyb-GAL4 / UAS-Gelsolin*

S4A and S4B Fig

Control: *nSyb-QF2, nSyb-GAL4, UAS-Atg8a-GFP / +*

gelsolin-OE (overexpression): *UAS-Gelsolin / nSyb-QF2, nSyb-GAL4, UAS-Atg8a-GFP*

cofilin-OE (overexpression): *UAS-cofilin / nSyb-QF2, nSyb-GAL4, UAS-Atg8a-GFP*

S4C-S4H Fig

Control: *nSyb-QF2, nSyb-GAL4 / +*

gelsolin-OE (overexpression): *UAS-Gelsolin / nSyb-QF2, nSyb-GAL4*

cofilin-OE (overexpression): *UAS-cofilin / nSyb-QF2, nSyb-GAL4*

S4I and S4J Fig

Control: *nSyb-QF2, nSyb-GAL4 / +*

gelsolin-OE (overexpression): *UAS-GFP-mCherry-Atg8a* / + ; *UAS-Gelsolin / nSyb-QF2, nSyb-GAL4*

cofilin-OE (overexpression): *UAS-GFP-mCherry-Atg8a* / + ; *UAS-cofilin / nSyb-QF2, nSyb-GAL4*

S5 Fig

Control: *UAS-mtKeima / + ; nSyb-QF2, nSyb-GAL4 / +*.

gelsolin-OE (overexpression): *UAS-mtKeima* / + ; *UAS-Gelsolin / nSyb-QF2, nSyb-GAL4*

cofilin-OE (overexpression): *UAS-mtKeima* / + ; *UAS-cofilin / nSyb-QF2, nSyb-GAL4*

S6 Fig

Control: *nSyb-QF2, nSyb-GAL4 / +*

gelsolin-OE (overexpression): *UAS-Gelsolin / nSyb-QF2, nSyb-GAL4*

cofilin-OE (overexpression): *UAS-cofilin / nSyb-QF2, nSyb-GAL4*

S7A and S7B Fig

α-syn, Arp2^f04069^/+: *Arp2^f04069^ / +; QUAS-wild type α-synuclein*, *nSyb-QF2, nSyb-GAL4, UAS-Atg8a-GFP / +*

α-syn, Arp3 RNAi #2: *UAS-Arp3^HMJ21357^* / + ; *QUAS-wild type α-synuclein*, *nSyb-QF2, nSyb-GAL4, UAS-Atg8a-GFP / +*

Arp2^f04069^/+: *Arp2^f04069^ / +; nSyb-QF2, nSyb-GAL4, UAS-Atg8a-GFP / +*

Arp3 RNAi #2: *UAS-Arp3^HMJ21357^* / + ; *nSyb-QF2, nSyb-GAL4, UAS-Atg8a-GFP / +*

S7C Fig

Control: *nSyb-QF2, nSyb-GAL4 / +*

α-synuclein: *QUAS-wild type α-synuclein*, *nSyb-QF2, nSyb-GAL4 / +*

α-syn, Arp2^f04069^/+: *Arp2^f04069^ / +; QUAS-wild type α-synuclein*, *nSyb-QF2, nSyb-GAL4 / +*

α-syn, Arp2 RNAi: *QUAS-wild type α-synuclein*, *nSyb-QF2, nSyb-GAL4 / UAS-Arp2^JF02785^*

α-syn, Arp3 RNAi #1: *QUAS-wild type α-synuclein*, *nSyb-QF2, nSyb-GAL4 / UAS-Arp2^HMS0071^*

α-syn, Arp3 RNAi #2: *UAS-Arp3^HMJ21357^* / + ; *QUAS-wild type α-synuclein*, *nSyb-QF2, nSyb-GAL4 / +*

α-syn, Arpc1 RNAi: *QUAS-wild type α-synuclein*, *nSyb-QF2, nSyb-GAL4 / UAS-Arpc1^JF01763^*
