# Supplementary material for: α-synuclein impairs autophagosome maturation through abnormal actin stabilization
Source: PLoS Genet. 2021 Feb 8;17(2):e1009359. doi: 10.1371/journal.pgen.1009359 (PMC7895402; doi:10.1371/journal.pgen.1009359)
Supplement: Attachment — Submitted filename: Response to reviewers.docx [file pgen.1009359.s010.docx]

Dear Dr. Copenhaver,

Thank you for forwarding the comments of the reviewers and giving us the opportunity to revise our manuscript. We appreciate the thoughtful comments of the reviewers and have prepared an edited manuscript, which addresses both of the reviewers’ concerns. A detailed response to the reviewers, whose comments are provided in full in italics, follows with our responses in plain text.

Sincerely yours,

Mel Feany

***Reviewer #1****: The manuscript explores the possibility that the expression of human alpha-synuclein stabilizes F-actin and a consequence impairs autophagosome maturation which in turn promotes dopaminergic cell loss.
To test this hypothesis they co-express with alpha-synuclein severing proteins gelsolin and cofilin in order to reduce F-actin levels in the cell. the expression of these two proteins revert several markers associated with the reduction of autophagosome activity.
The manuscript is clearly written and should be published however there are two minor concerns that should be addressed before publication.*

*1) Labels in immunofluorescent panels figure 1 are hard to read. Figure 1 images are of very low quality.*

Our apologies for the low quality of the image provided in the review PDF. Figure 1 is very large and we were only able to upload a low resolution version for the review PDF. By clicking on the link provided a high-resolution version is available, which we believe addresses the concern of the labels being visible as well as the quality of the images.

*2) Green signal in figure 2 is overly saturated, why? What were the acquisition conditions? It appears that there is practically no dynamic range in the green channel.*

The green channel appears overly saturated because we increased the intensity of both the control and alpha-synuclein images equally to allow visualization of the modest signal in controls. We appreciate the reviewer’s point and have now provided lower intensity panels for Figure 2A. We have also significantly expanded the section detailing our acquisition parameters and image processing procedures. Specifically, controls were performed to ensure that signals were not saturated and the intensities of all images were modified in an identical manner across genotypes.

***Reviewer #2****: Summary: The manuscript by Sarkar et al., is a follow up study based on the development of a well-validated Drosophila model (Ordonez et al., Neuron, 2018) for α-synuclein neuropathies including the formation of so-called Lewy bodies. In that previous study, the Feany lab reported that reduced mitochondrial function was linked to abnormal actin-mediated processes but the mechanisms underlying these defects remained to be established. Cellular defects characteristic of the human neuropathies were reproduced in Drosophila neurons expressing a disease-inducing version of the human α-synuclein gene. In the current manuscript, the authors take a significant step forward in delineating the mechanism underlying this actin-based pathology by identifying a stabilizing role of the actin cytoskeleton in blocking a key step in autophagy. They use a range of well crafted tools including antibody staining, expression of compartment-specific markers (some of which change output in response to differing cellular compartments), and over-expression of actin severing proteins (gelsolin or cofilin) to analyze in detail the process of autophagy in neurons expressing the disease associated form of α-synuclein. The authors find that the autophagy, in particular the fusion of autophagosomes to the lysosomal compartment, is greatly reduced in neurons of α-synuclein flies and show further that reduction of key elements of the actin remodeling system (Arp2,3) can greatly ameliorate this condition at the cellular level. These results point the way to potential future therapies (e.g., with rapamycin) for treating α-synucleinopathies by targeted clearing of actin-tethered autophagosomes, potentially by promoting autophagic clearance of α-synuclein itself.

Critique: Experiments presented in this study are of high quality and support the authors’ conclusions and interpretations of the data. These findings of are of broad interest and represent a substantial advance in the field by delineating an actin-dependent block in autophagy as the key cellular defect in response to expression of a disease associated form of α-synuclein. Based on the quality of the work and its impact, I believe this manuscript merits publication in PLOS Genetics and recommend publishing this paper without any additional required experiments. A few suggestions that the authors may wish to consider in formulating the final version of the manuscript are included below:*

*1) An interesting observation that the authors make in the context of examining the rescuing effects of over-expressing actin severing proteins is that the size of Atg8a-GFP expressing vesicles is decreased with only minor if any effects on the number of these structures (Fig. 3A). The effect is also seen to some degree with the p62 and Arl8 markers (Fig. 3C,E). The authors note this effect in the text and comment reasonably that experimental analysis of the basis for this primary effect on autophagosome size rather than number is beyond the scope of the current study. I agree that no new experiments need to be performed, however, it might be helpful for the reader to understand what the possible basis for this specific effect could be. For example, might this finding suggest that rather than nucleating the initial formation of excess autophagosomes the actin-dependent effect of α-synuclein is primarily involved in a feedback mechanism that keeps adding membrane to nascent autophagosomes (perhaps a non-actin-dependent mechanism triggers their initial formation?). Is such a two-step self-amplifying model tenable and if so how might the action of the disease form of α-synuclein be involved? Also, could there be a connection between the formation of the cellular inclusions (e.g., shown in Fig. 1I) and such a positive feedback cycle? Could accretion of such a mass of complex membrane impede fusion with the lysosomal compartment?*

We do not currently have a clear understanding of the mechanistic basis for the enlargement of the autophagosomes that we observe. Lysosomal enlargement has been associated with enhanced lysosomal activity as well as with lysosomal dysfunction due to multiple types of pathology. A review of the literature suggests that the same is likely to be true for enlargement of autophagosomes. Enlargement of autophagosomes can occur as a consequence of defective fusion with lysosomes (Wang et al., 2015; Nakamura and Yoshimori, 2017), but has also been reported in conditions with apparently normal fusion as well (Mauvezin et al., 2015), suggesting that enlargement may be a nonspecific feature of autophagosomal dysfunction. Thus, although the interesting mechanism suggested by the reviewer might underlie the enlargement of autophagosomes, there are other possibilities as well and we believe that it would be most prudent not to speculate further at this time.

*2) The authors show convincing evidence that RNAi of components of the Arp2,3 complex greatly reduce the cellular effects of α-synuclein. Does this cellular rescue also translate to rescue in longevity studies (or other organism level phenotypes)? If not, perhaps the authors could state that and offer a potential explanation?*

The reviewer raises an interesting point. As reported in Ordonez et al. (2018, Figure S1C), there is only a modest reduction in lifespan in alpha-synuclein transgenic flies. We did not attempt to modify lifespan in these studies because the substantial influence of genetic background on lifespan would make these studies challenging to perform in a highly rigorous manner. We agree that assessment of other organism-level phenotypes would be of interest but would be a significant additional effort. We also suspect that interpretation of such studies might be challenging. As can be seen in Figure 8E,F, genetic alteration of Arp2/3 function influences the number of lysosomes even in the absence of transgenic human alpha-synuclein expression. These findings, and the known role of the Arp2/3 complex in various aspects of cell biology, suggest that interpretation of effects on organism-level phenotypes may be complex.

*3) Also regarding the suppression of the α-synuclein phenotype by reduction in Arp2,3 complex members, does heterozygosity for mutant loss-of-function alleles of these same genes (Arp2, Arp3 Arpc1) also result in suppression of cellular α-synuclein phenotypes? Along the same lines, is there any evidence from GWAS studies in humans that heterozygosity for Arp2,3 components reduce the probability of developing α-synucleinopathies or that amplification of any these loci (e.g., by copy number variation or local duplications) score as a risk factor?*

As mentioned above in response to point 2), we did not comprehensively assess organism-level phenotypes Arp2/3 modified flies, which would be a substantial effort and might be challenging to interpret (see Figure 8E,F). We agree that our findings suggest that genetic variants in actin cytoskeletal proteins could modulate risk of Parkinson’s disease in patients. Although to our knowledge Arp2/3 family members have not been identified in genetic studies in Parkinson’s patients, proteins encoded by two loci identified through GWAS in Parkinson’s disease, *BIN3* and *TAU*, have both been implicated in actin cytoskeletal regulation. GWAS studies in Parkinson’s disease continue to expand and may reveal more evidence for a role of the actin cytoskeleton in the future.


*4) A small stylistic point. In my view, it is best not to begin sentences with a preposition (e.g., To determine how….). I know that this style has now become accepted practice, but I personally find it to be a repetitious form that can readily be avoided. For example, the following more active rephrasing would be preferable in my humble opinion:

Current sentence: To detect autophagosomes, we crossed flies containing the transgenic reporter construct UAS-Atg8a-GFP to α-synuclein transgenic flies and visualized tagged Atg8a by immunofluorescence in brain sections.

Alternative phrasing: We detected autophagosomes by crossing flies containing the transgenic reporter construct UAS-Atg8a-GFP to α-synuclein transgenic flies and visualized tagged Atg8a by immunofluorescence in brain sections.*

Thank you for taking the time to provide the suggestion. We have edited the sentence indicated and a number of others beginning with a preposition. We always appreciate an opportunity to improve our writing skills.

Mauvezin, C., P. Nagy, G. Juhász, and T.P. Neufeld. 2015. Autophagosome-lysosome fusion is independent of V-ATPase-mediated acidification. *Nat Commun*. 6:7007. doi:10.1038/ncomms8007.

Nakamura, S., and T. Yoshimori. 2017. New insights into autophagosome-lysosome fusion. *J. Cell. Sci.* 130:1209–1216. doi:10.1242/jcs.196352.

Wang, H., H.-Q. Sun, X. Zhu, L. Zhang, J. Albanesi, B. Levine, and H. Yin. 2015. GABARAPs regulate PI4P-dependent autophagosome:lysosome fusion. *Proc. Natl. Acad. Sci. U.S.A.* 112:7015–7020. doi:10.1073/pnas.1507263112.
